# Surveillance of healthcare-associated infections in a neonatal intensive care unit in Italy during 2006–2010

**DOI:** 10.1186/s12879-015-0909-9

**Published:** 2015-03-25

**Authors:** Valeria Crivaro, Lidija Bogdanović, Maria Bagattini, Vita Dora Iula, Mariarosaria Catania, Francesco Raimondi, Maria Triassi, Raffaele Zarrilli

**Affiliations:** Department of Public Health, University of Naples “Federico II”, Naples, Italy; AORN dei Colli, Monaldi Hospital, Via L. Bianchi, Naples, Italy; Department of Molecular Medicine and Medical Biotechnologies, University of Naples “Federico II”, Naples, Italy; Division of Neonatology, Department of Medical Translational Sciences, University of Naples “Federico II”, Naples, Italy; Italian Study Group of Hospital Hygiene, Italian Society of Hygiene, Preventive Medicine and Public Health, (GISIO), Rome, Italy

**Keywords:** Neonatal intensive care units, Healthcare-associated infections, Active surveillance

## Abstract

**Background:**

Healthcare-associated infections (HAIs) are a frequent complication associated with hospitalization of infants in neonatal intensive care units (NICUs). The aim of this study was to evaluate and describe the results of surveillance of HAIs in a III level NICU in Naples, Italy during 2006–2010.

**Methods:**

The surveillance covered 1,699 neonates of all birth weight (BW) classes with > 2 days NICU stay. Infections were defined using standard Centers for Disease Control and Prevention definitions adapted to neonatal pathology and were considered to be healthcare-associated if they developed > 2 days after NICU admission.

**Results:**

One hundred-fifty-three HAIs were diagnosed with a frequency of 9% and an incidence density of 3.5 per 1000 days of hospital stay. HAIs developed in all BW classes, but patients weighing ≤ 1000 g at birth were more affected with a decreasing trend from the lowest to the highest BW classes. Sepsis proved to be the most frequent infection (44.4%), followed by urinary tract infection (UTI) (28.8%), pneumonia (25.5%) and meningitis (1.3%). Device associated infections (i.e. central line-associated bloodstream infections (BSIs), umbilical catheter-associated BSI and ventilator associated pneumonias (VAPs) represented 64.1% of all HAIs. Most frequent pathogens responsible for all types of infections were: *P. aeruginosa* (17%), *C. parapsilosis* (16.3%), *E. coli* (13.1%), *C. albicans* (10.5%), non- extended spectrum beta-lactamase (ESBL) *K. pneumoniae* (7.8%), and coagulase-negative Staphylococci (5.2%). No microbiological diagnosis was achieved for 6.5% of infections.

**Conclusions:**

HAIs developed in all BW classes but low BW neonates were at major risk to acquire HAIs in our NICU. Use of central line-, umbilical-catheter and mechanical ventilation was associated with higher risk of infection. Our findings highlight the importance of an extensive surveillance approach in the NICU setting, which includes all BW classes of neonates and monitors infections associated with the use of medical devices.

## Background

Healthcare-associated infections (HAIs) are frequent and critical complications associated with hospitalization of neonates, especially very low birth weight (VLBW) neonates, in neonatal intensive care units (NICUs) [1,2]. Active surveillance systems for HAIs in NICUs have been developed in USA and Canada by the National Healthcare Safety Network (NHSN) [3,4], the Vermont Oxford Network [5,6] and the Canadian Neonatal Network [7]. In Europe, surveillance systems for HAIs in NICUs are active in Germany [8,9] and in England [10].

The first national report on HAIs in Italian intensive care units (ICUs) has recently been published [11]. The document shows the results of HAIs surveillance activities for the 2009–2010 period and mainly focuses on adult ICUs, with a very limited contribution of pediatric ICUs and none of NICUs.

While several studies have shown the spread of specific nosocomial pathogens, such as ESBL producing Enterobacteriaceae [12,13], *Pseudomonas aeruginosa* [14], methicillin resistant *Staphylococcus aureus* [15], extensively drug resistant (XDR) *Acinetobacter baumannii* [16], *Candida parapsilosis* [17] in Italian NICUs, few studies were performed on the prevalence [18] or incidence of HAIs in NICUs [19-21]. Active surveillance of HAIs was utilized in the majority of the studies from Italian NICUs [12-16,18,21].

Objective of the present study was to describe and discuss the results of surveillance of HAIs in a neonatal intensive care unit in Italy during 2006–2010 using the NHSN surveillance protocol.

## Methods

### Setting

The University of Naples “Federico II” NICU is a III level NICU with a total of 25 incubators and cradles. The ward serves the University Obstetric Clinic (approximately 2000 births/year) which is both a high risk pregnancy center and an obstetric emergency service. Moreover, the NICU admits outborn neonates from the regional Newborn Emergency Transport Service.

### Active surveillance

Healthcare-associated infections (HAIs) active, patient-based surveillance (AS) is continuously carried out by trained personnel. Data are collected on weekly basis directly from patient’s charts. Any clinical issues are directly discussed with caregivers. Data are analyzed on monthly basis and expressed as monthly report. Monthly report consist of patient data, data on swab isolations of sentinel pathogens, device utilization ratios and infection data. All neonates with > 2 days NICU stay enter the AS system and data regarding birth (birth weight (BW), gestational age, type of delivery, and Apgar score), invasive device exposure (days of umbilical and central line catheterization and of invasive ventilation), antimicrobial therapy exposure (yes/no) and infections are collected. End of surveillance coincides with neonates’ discharge from the ward. Infections are defined using standard Centers for Disease Control and Prevention definitions adapted to neonatal pathology [22-24] and are considered to be healthcare-associated if they develop > 2 days after NICU admission. We exclude neonates with a vertical infection (ie, an infection transmitted from mother to child) or infection acquired during delivery. This paper analyzes data from the HAIs AS system over a 5 years long period (2006–2010). For this study purposes, only sepsis, meningitis, pneumonia, and urinary tract infections (UTIs) were considered. Central line-associated blood-stream infections (BSI), umbilical catheter-associated BSI and ventilator-associated pneumonia (VAP) were attributed if a central line, an umbilical catheter and invasive ventilation, respectively, were in place at the time of or within 48 hours before infection onset [24]. Frequency measures were calculated as percent of infection and as incidence densities, i.e. infection rates per 1000 days of hospital stay or 1000 days of directly related invasive device within 5 BW classes (≤750 g, 751–1000 g, 1001–1500 g, 1501–2500 g, and ≥ 2501 g). Device utilization rates within such classes were also calculated. The etiology of all infections within each birthweight class was assessed during the study period.

HAIs surveillance is regulated by the Regional Health Authority and it is one of the basic components of the Regional Plan for Healthcare-associated infections Prevention and Control [25]. The HAIs AS system manages data in a cumulative form with the ultimate goal of diseases’ surveillance in the population and continuous improvement of quality in healthcare. Results of surveillance activities are periodically reported to the Regional Health Authority. As such, no patient informed consent, nor local Ethical Committee authorization is required.

### Statistical analysis

Data were analysed using SPSS 11.0 (Chicago, IL, USA). Finally, ANOVA or Kruskal Wallis test were used as appropriate to assess significant differences (p < 0.05) in the above-mentioned variables over the five-years study period. Linear regression analysis was performed to detect significant time-dependent and BW-related trends as previously described [26,27].

## Results

Between years 2006 and 2010 1699 neonates, corresponding to 91.49% of all admissions to the ward, entered the HAIs AS system and a total of 43447 patient days were registered. The remaining 8.51% of admissions was excluded because either ineligible (length of stay < 2 days) or missed by the HAIs AS system. During the study period neonates with > 1000 g BW accounted for 74% of total patient days (1001–1500 g, 1501–2500 g, and ≥ 2501 g classes representing 26.7%, 28.1%, and 19.2%, respectively) while the remaining 26% was distributed among extremely low BW (ELBW) infants (≤750 g and 751–1000 g BW classes accounting for 9.1% and 16.9% of total patient days, respectively). Statistically significant differences in length of hospitalization over the five-years period were detected in the lower BW classes (p = 0.003, 0.047, and 0.004 for ≤ 750 g, 751–1000 g, and 1001–1500 g classes, respectively), and total patient days in the ≤ 750 g BW class showed a statistically significant decreasing trend over time (R^2^ = 0.228, p = 0.000) (Table 1).

**Table 1:** **Neonates data per birthweight category during the study period**

**Year**	**2006**	**2007**	**2008**	**2009**	**2010**	**Total**
**Hospitalized patients**	339	392	395	427	304	1857
**Surveilled patients**	310	357	357	384	291	1699
Number of patient days*	**8020**	**9012**	**9259**	**8361**	**8795**	**43447**
≤ 750 g	1127	890	903	475	564	3959
751-1000 g	1939	1194	1400	1055	1773	7361
1001-1500 g	1628	2748	2927	2258	2038	11599
1501-2500 g	2084	2585	2292	2731	2500	12192
>2500 g	1242	1595	1737	1842	1920	8336
Number of umbilical catheter days*	**729**	**803**	**775**	**924**	**1001**	**4232**
≤750 g	92	94	80	124	112	502
751-1000 g	180	74	106	88	217	665
1001-1500 g	191	280	265	259	267	1262
1501-2500 g	173	182	166	267	254	1042
>2500 g	93	173	158	186	151	761
Number of central line days*	**1044**	**1049**	**1374**	**1133**	**1395**	**5995**
≤750 g	213	194	214	125	145	891
751-1000 g	430	213	268	187	370	1468
1001-1500 g	187	263	439	396	402	1687
1501-2500 g	158	229	271	287	331	1276
>2500 g	179	150	182	138	147	796
Number of ventilator days*	**1044**	**843**	**1075**	**702**	**1544**	**5208**
≤750 g	298	286	362	149	258	1353
751-1000 g	492	245	214	77	604	1632
1001-1500 g	142	97	257	115	334	945
1501-2500 g	70	142	156	196	208	772
>2500 g	42	73	86	165	140	506

*Data refer to surveilled patients.

Percentiles of device utilization rates in the five BW classes are shown in Table 2. No statistically significant differences within each BW class were found over the five years study period for devices utilization rates, except for mechanical ventilation in the 751–1000 g and 1001–1500 g BW classes (p = 0.000 and 0.002, respectively). The latter showed an increasing trend over the studied interval (R^2^ = 0.091, p = 0.020).

**Table 2:** **Percentiles of the distribution of device utilization rates in surveilled neonates during the study period**

	**10°**	**25°**	**50°**	**75°**	**90°**
Umbilical catheter utilization rate					
≤750 g	.000	.021	.105	.191	.447
751-1000 g	.000	.029	.075	.125	.169
1001-1500 g	.052	.058	.105	.142	.178
1501-2500 g	.021	.049	.080	.117	.149
>2500 g	.017	.041	.081	.135	.167
Central catheter utilization rate					
≤750 g	.000	.038	.164	.395	.563
751-1000 g	.000	.085	.159	.302	.380
1001-1500 g	.017	.066	.140	.200	.277
1501-2500 g	.000	.030	.103	.161	.213
>2500 g	.000	.037	.074	.139	.221
Mechanical ventilation utilization rate					
≤750 g	.000	.117	.352	.564	.748
751-1000 g	.000	.059	.175	.329	.500
1001-1500 g	.005	.015	.035	.131	.190
1501-2500 g	.006	.020	.051	.102	.152
>2500 g	.000	.014	.039	.101	.137

The overall number of HAIs registered during the study period by the local AS system was 153 (9% total infection rate and 3.5 total incidence density per 1000 days of hospital stay). HAIs developed in all BW classes, but patients weighing ≤ 1000 g at birth were more affected (45.8% of all HAIs) and a statistically significant decreasing trend (R^2^ = 0.964, p = 0.003) from the lowest to the highest BW classes in terms of incidence density per 1000 days of hospital stay was observed (7.83, 5.298, 3.104, 2.297, and 2.279 in ≤ 750 g, 751–1000 g, 1001–1500 g, 1501–2500 g, and ≥ 2501 g BW newborns, respectively). On the whole, sepsis proved to be the most frequent infection (44.4%), followed by UTIs (28.8%), pneumonia (25.5%) and meningitis (1.3%). Device associated infections (i.e. central line-associated BSI, umbilical catheter-associated BSI and VAPs) represented 64.1% of all HAIs. Incidence densities of device-associated infections among the five BW classes are shown in Table 3. Incidence densities of the three types of device associated infections showed no statistically significant differences within each BW class over the five years study period, and within the five years of the study period when related to the five BW classes, with the exception of ventilator-associated pneumonia (VAP) in year 2008, which showed a statistically significant, decreasing trend from the lowest to the highest BW classes (R^2^ = 0.130, p = 0.005).

**Table 3:** **Incidence densities of device-associated infections per birth weight category in surveilled neonates**

**Device-associated infection**	≤**750 g**	**751-1000 g**	**1001-1500 g**	**1501-2500 g**	**>2500 g**
Umbilical catheter associated bloodstream infection (pooled mean)*	6.127	0.331	1.231	0.892	1.695
Central catheter associated bloodstream infection (pooled mean)*	11.636	8.644	4.731	4.544	6.981
VAP (pooled mean)*	9.93	6.896	1.346	1.577	5.595

*Data refer to cases per 1000 specific device-days.

Etiology of device-associated infections among the five BW classes are shown in Table 4. Most frequent pathogens responsible for all types of infections were: *P. aeruginosa* (17%), *C. parapsilosis* (16.3%), *E. coli* (13.1%), *C. albicans* (10.5%), non-ESBL *K. pneumoniae* (7.8%), and coagulase-negative Staphylococci (5.2%). The remaining 23.5% of infections were caused by either Enterobacteriaceae, *Candida* species, *S. aureus* or other gram-positive cocci, each accounting for less than 5% of all infections. No statistically significant differences during the five-years period were detected among such pathogens. Moreover, for 6.5% of infections no microbiological diagnoses were achieved; such cases showed statistically significant differences during the five-years period (p = 0.024) and a statistically significant decreasing trend over the studied interval (R^2^ = 0.116, p = 0.008).

**Table 4:** **Etiology of device-associated infections per birth weight category in surveilled neonates**

**Umbilical catheter associated bloodstream infection**	**≤750 g**	**751-1000 g**	**1001-1500 g**	**1501-2500 g**	**>2500 g**	**Total (%)**
*Candida parapsilosis*	3	1	2	0	0	6 (50)
*Escherichia coli*	0	0	0	2	1	3 (25)
*Candida albicans*	1	0	0	0	1	2(16.7)
CONS	0	1	0	0	0	1 (8.3)
Total (% within BW category)	4 (33.3)	2 (16.7)	2 (16.7)	2 (16.7)	2 (16.7)	12 (100)
**Central catheter associated bloodstream infection**	**≤750 g**	**751-1000 g**	**1001-1500 g**	**1501-2500 g**	**>2500 g**	**Total (%**)
*Candida parapsilosis*	3	4	5	2	3	17 (35.4)
*Candida albicans*	1	3	0	2	1	7 (14.6)
*Escherichia coli*	0	4	0	0	0	4 (8.3)
*Pseudomonas aeruginosa*	3	1	0	0	0	4 (8.3)
*Klebsiella pneumoniae* ESBL+	0	0	1	2	0	3 (6.3)
*Serratia marcescens*	2	1	0	0	0	3 (6.3)
CONS	0	0	2	0	0	2 (4.2)
*Candida glabrata*	0	0	1	0	0	1 (2.1)
Enterobacter spp.	0	1	0	0	0	1 (2.1)
*Enterococcus faecalis*	0	0	1	0	0	1 (2.1)
Not determined	0	0	1	0	0	1 (2.1)
polimicrobial (*Candida albicans* + *Pseudomonas aeruginosa*)	1	0	0	0	0	1 (2.1)
*Rothia mucillaginosa*	0	0	0	0	1	1 (2.1)
*Staphylococcus aureus*	0	0	0	1	0	1 (2.1)
*Streptococcus* spp.	1	0	0	0	0	1 (2.1)
Total (% within BW category)	11 (22.9)	14 (29.2)	11 (22.9)	7 (14.6)	5 (10.4)	48 (100)
**VAP**	**≤750 g**	**751-1000 g**	**1001-1500 g**	**1501-2500 g**	**>2500 g**	**Total (%)**
*Pseudomonas aeruginosa*	5	7	0	1	2	15 (41.7)
*Acinetobacter baumannii*	2	1	1	0	0	4 (11.1)
CONS	0	2	1	0	1	4 (11.1)
*Klebsiella pneumoniae* ESBL+	1	2	1	0	0	4 (11.1)
*Candida albicans*	1	1	0	0	0	2 (5.6)
*Escherichia coli*	1	1	0	0	0	2 (5.6)
Not determined	1	0	0	1	0	2 (5.6)
Polimicrobial (*K. pneumoniae* ESBL+ + *P. aeruginosa*)	0	0	0	1	0	1 (2.8)
*Staphylococcus aureus*	0	1	0	0	0	1 (2.8)
*Stenotrophomonas maltophilia*	0	1	0	0	0	1 (2.8)
Total (% within BW category)	11 (30.6)	16 (44.4)	3 (8.3)	3 (8.3)	3 (8.3)	36 (100)

*Abbreviations:* NICU, neonatal intensive care unit; CONS, coagulase negative staphylococci; ESBL+, extended spectrum beta-lactamase producer; VAP, ventilator-associated pneumonia.

The most frequent device-unrelated HAIs were UTIs (77.2% of all), which mainly affected neonates belonging to the 1001–1500 g and 1501–2500 g BW classes (distribution of UTIs was 0%, 9,1%, 45.5%, 31.8% and 13,6% in ≤ 750 g, 751–1000 g, 1001–1500 g, 1501–2500 g, and ≥ 2501 g BW newborns, respectively). UTIs incidence density per 1000 days of hospital stay showed no statistically significant differences within each BW class over the five years study period, and within the five years of the study period when related to the five BW classes, with the exception of year 2008, which showed a statistically significant increasing trend from the lowest to the highest BW classes (R^2^ = 0.812, p = 0.037).

Twenty-five percent of UTIs were caused by *E. coli*, followed by *K. pneumoniae* (18.2%, half of which displayed ESBL phenotype), *Candida spp*. (15.9%), *P. aeruginosa* (13,6%), *Enterobacter spp*. and *Enterococcus spp*. (6.8% each); the remaining 13.7% included ESBL-producing *Klebsiella oxytoca* and *Serratia marcescens*, *Proteus mirabilis*, polimicrobial and undetermined aetiologies.

One half of catheter-unrelated sepsis was not supported by microbiological identification and one half was caused by *C. albicans*, *E. faecalis*, *P. aeruginosa*, and *Streptococcus viridans*. Pathogens responsible for the 3 ventilator-unassociated pneumonias were *S. epidermidis*, *E. coli* and *K. pneumoniae*. In one case, meningitis evolved from a *C. albicans* umbilical catheter-associated BSI in a ≤ 750 g BW neonate, in the other no aetiology was defined (Table 4).

## Discussion

Surveillance is a well-established prevention measure of healthcare-associated infections (HAIs) and reductions in HAIs rates are to be expected on the medium to long term period at those institutions which systematically carry out such activities [23,28,29]. HAIs AS in the University of Naples “Federico II” NICU was started in the mid 1990s and has been going on since then according to the Atlanta’s CDC methodology (National Nosocomial Infections Surveillance System NNIS before and NHSN nowadays) [22-24]. Data here described show that during the 2006–2010 period no significant change in HAIs rates took place. Nevertheless, when compared to previous data referred to the same NICU over a 54-months period between years 1996 and 2000 [12], our findings demonstrate that HAIs rates, in terms of both total infection rate and total incidence density per 1000 days of hospital stay, were reduced by nearly half in a fourteen years time span (9% and 3.5 vs. 17.2% and 7.3). Further analysis of factors which, other than HAIs AS, led to such variations goes beyond the scope of this research.

Moreover, during the study period here described, two outbreaks [14,16] took place in the ward and this may partially explain the absence of statistically significant changes in all HAIs rates. On the other hand, during one of such outbreaks [14], an intense educational programme on hand hygiene was carried out in the ward and its positive effects may have improved infection rates.

The HAIs AS system here adopted is basically inspired to Centers for Disease Control and Prevention (CDC)’s NHSN - device associated module, thus benchmarking of our data against infection rates of NHSN reports appears to be methodologically correct. For this purpose, NHSN data reports referred to the 2006–2009 period were considered, while the one referred to year 2010 was excluded because starting from that year bloodstream infections returned to be related to central lines in general, with no distinction between umbilical and central catheters [3,4,30].

Pooled mean infection rates at our institution proved to be higher than those reported by NHSN, yet always lower than the 90° value for the middle BW classes (751–1000 g, 1001–1500 g, and 1501–2500 g). On the contrary, extreme BW classes (≤750 g and ≥ 2501 g) infection rates constantly performed as high outliers. Such results are not in contrast with the strong, BW-related decreasing trend (R^2^ = 0.964, p = 0.003) we observed from the lowest to the highest BW classes, since it refers to the overall infection incidence density per 1000 days of hospital stay and not only to device-associated infections. Moreover, with the exception of umbilical catheter utilization rate in ≤ 750 g BW neonates, our percentile distributions of devices’ utilization rates show lower values than those of NHSN. These findings suggest that specific problems may exist in the ≤ 750 g and ≥ 2501 g BW classes, but they also point out that comparisons between very diverse healthcare systems may be confounding or not appropriate because of objective differences in clinical practice and standards of care.

A well-established European neonatal surveillance network is Germany’s Neonatal-Krankenhaus Infektions Surveillance System (NEO-KISS). Unlike the NHSN surveillance system, the NEO-KISS system monitors HAI occurring in all very low birth weight (VLBW) infants (BW < 1500 g) in NICUs until discharge, until death, or until they reach 1800 g. The system identifies different BW classes (≤499 g, 500–999 g, 1000–1499 g) and makes no distinction between umbilical and central line [8,9]. Thus, a possible comparison between our data and the latest NEO-KISS report [31] can only be made for VAP rates in the 1000–1500 BW class, which appear to be similar (1,34 and 1,17 at our NICU and in NEO-KISS, respectively).

A more pertinent benchmarking would be on a national scale. As previously pointed out, though, no data from NICUs is included in the first national report on HAIs in Italian ICUs. A multicentre prospective cohort study in six Italian NICUs lasting 28 months described a 12.8% infection rate, with an incidence density of 6.93 episodes per 1,000 patient days [21]. A three years surveillance report from a single NICU showed similar results: 13.2% infection rate with an incidence density of 7.8 episodes per 1,000 patient days [20]. Our data appear to be considerably lower but, again, questions on the accuracy of national-based, yet network-unrelated comparisons arise.

The present study shows that over time a substantial change in HAIs etiology at our NICU has taken place: in the previous decade the most commonly isolated microorganism was *Staphylococcus epidermidis*, which caused 28% of all HAIs [12]. During the 2006–2010 period, most frequent pathogens were gram-negative bacteria *(P. aeruginosa*, *E. coli, K. pneumoniae)* and fungi *(C. parapsilosis*, *C. albicans)*, while coagulase-negative Staphylococci dropped down to 5.2%. Such trend proved to be stable over time (no statistically significant differences during the five-years period were detected among such pathogens) and quite well reproducible in all specific types of HAIs (umbilical and central catheter-associated BSIs, VAPs and UTIs). Moreover, it is worth-mentioning that BSIs at our institution were mainly caused by *C. albicans* and *C. parapsilosis*. Fluconazole prophylaxis for neonates ≤1500 g BW was introduced during 2009 at our NICU. These findings partially agree with a previous prospective surveillance study on nosocomial infections in six NICUs in Brazil which showed that Gram-negative rods (mainly *Klebsiella* sp. and *E. coli*) were responsible for 51.6% episodes of BSI, Gram-positive organisms and Candida sp. accounted for 37.4 and 11.1%, respectively [32]. On the other hand, our data strongly differ from two recent NICU long period-based reports describing a prevalent gram-positive etiology for BSI [33,34]. These discrepancies may depend on differences in the clinical settings.

Our data also showed that 48/60 (80%) BSI were associated with central-line catheter use, 12/60 (20%) with umbilical catheter. Although data collection is more complicated, we speculate that the separation between umbilical and percutaneous catheters-associated BSIs is worth maintaining in order to better identify problems and prevention strategies.

Interestingly, clinical sepsis (i.e. not determined etiology) significantly decreased during the study period. This should imply less broad spectrum molecules use and targeted antimicrobial prescription, however such variables are not examined in this paper and the considered time interval is probably too short to show variations in bacterial resistance phenotypes.

Most reports on HAIs in NICUs focus on BSIs and, eventually, on VAPs. Our data shows that the most frequent infection after BSIs is UTI. Although UTI were diagnosed according to validated criteria [4], no sterile urine collection through supraumbilical aspiration was performed. In agreement with our data, a previous surveillance study of HAI in an Italian NICU reported that UTI are the third most frequent cause of infection after BSI and pneumonia [20].

Finally, the data reported herein show that HAIs developed in all BW classes. Although a decreasing trend from the lowest to the highest BW classes in terms of incidence density per 1000 days of hospital stay was observed, all device-associated infection rates in ≥ 2501 g BW neonates proved to be higher than those recorded in the 1000–2500 g BW classes. This is in agreement with other surveillance studies in European NICUs which have also described infectious complications in the upper BW classes [10,20,21,33]. Moreover, this finding underlines the importance of HAIs surveillance in NICUs for all BW classes newborns.

## Conclusions

In our NICU, all BW classes were affected by HAIs, but low birth weight neonates were at major risk to acquire HAIs. Sepsis, UTI and pneumonia were the most frequent infection in our NICU. Because use of central line-, umbilical-catheter and mechanical ventilation was associated with higher risk of HAI in our clinical setting, a surveillance protocol which monitors device-associated infections in all BW classes neonates may represent a useful tool to prevent HAIs in the NICU. The absence of national, network-based data in the NICU context impairs benchmarking activities and, thus, may limit surveillance systems’ contribution to HAIs prevention.

## Abbreviations

HAIs: Healthcare-associated infections
NICU: Neonatal intensive care
AS: Active surveillance
UTI: Urinary tract infection
BSI: Bloodstream infection
VAP: Ventilator associated pneumonia
BW: Birth weight
VLBW: Very low birth weight
ELBW: Extremely low BW
CONS: Coagulase negative staphylococci
ESBL: Extended spectrum beta-lactamase
XDR: Extensively drug resistant
CDC: Centers for Disease Control and Prevention
NHSN: National Healthcare Safety Network
NNIS: National Nosocomial Infections Surveillance System
NEO-KISS: Neonatal-Krankenhaus Infektions Surveillance System
